# Ultrasound guided percutaneous microwave ablation of benign breast lesions

**DOI:** 10.18632/oncotarget.18123

**Published:** 2017-05-23

**Authors:** Jie Yu, Bao-Hua Chen, Jing Zhang, Zhi-Yu Han, Han Wu, Yan Huang, Meng-Juan Mu, Ping Liang

**Affiliations:** ^1^ Department of Interventional Ultrasound, Chinese PLA General Hospital, Beijing, China; ^2^ Department of General Surgery, The People’s Liberation Army One Eight Four Hospital, Yingtan, China

**Keywords:** breast, benign, microwave ablation, ultrasound, volume reduction

## Abstract

The benign breast lesions (BBLs) share a high incidence for women and therapy methods with minimal invasion and better cosmetic

outcome are thirsted for. In this study, 122 patients with 198 biopsy-proved BBLs were enrolled. Ultrasound (US)-guided microwave ablation (MWA) was performed with local anesthesia from November, 2013 to April, 2016. The mean longest tumor size assessed was 1.6±0.7 cm (ranging 0.7-4.9 cm). MWA was successfully performed in all cases including 85 lesions adjacent to the skin, pectoralis and areola. The mean ablation time was 3.2mins (ranging 0.5-18.3 mins). 99.5% of BBLs showed complete ablation when assessed by magnetic resonance imaging and 100% of them by US. At the median 14-month follow-up, the BBLs were not palpable in 45.9 % of the cases (palpable in 90.2 % of the cases before MWA) and the mean volume reduction ratio was 78.4±33.5% for total lesions and 89.3±20.8%, 84.7±27.6% and 55.9±32.9% for ≤1.0 cm, 1.1-2.0cm and >2.0 cm lesions in 12-month follow-up, respectively. Cosmesis were reported as good or excellent in 100 % by physician and patients. No side effect was found. The MWA of the BBLs proved feasible and effective, while showing meaningful reduction in volume, palpability and cosmetic satisfying outcomes.

## INTRODUCTION

Benign breast lesions (BBLs) are the outcomes of proliferation of ductal or lobular tissue that are manifested by the presence of palpable lumps or masses [1-3]. The BBL shares a high incidence for women and it should deserve attention for its high prevalence, its increasing size, its impact on women’s quality of life, and its cancerous potential for some histologic types [1, 2]. Although the natural history would suggest the BBL diagnosed with minimally invasive needle core biopsy can be safely observed, half million of symptomatic BBLs are still surgically removed or vacuum-assisted biopsy per year [2-4]. With the thirst for therapy method with minimal invasion and better cosmetic outcome, alternative techniques are currently in development for the BBLs as cryoablation, radiofrequency ablation (RFA), microwave ablation (MWA), high-intensity focused ultrasound (HIFU), and laser ablation [5-8]. Compared with vacuum-assisted biopsy, ablation shares advantages including treating multiple tumors simultaneously and not being limited by larger tumors size. But different from the wide application in the liver, ablation techniques are relatively new minimally-invasive treatments for breast masses with relatively limited studies, especially for the BBLs.

While as a promising thermal ablation technique—, MWA has several theoretical advantages over other ablation techniques in producing consistently higher intra-tumor temperatures, larger ablation volumes, less ablation time, less dependence on the electrical conductivities of tissue and energy delivery less limited by the exponentially rising electrical impedance of BBL tissue [9, 10]. Therefore MWA may have higher potential for complete destruction of focal breast mass, which was verified by *ex vivo* experimental study [10]. However, as a relatively new technique, only very limited studies described the initial results of MWA for treating breast masses [11-13]. Therefore, we performed this study to prospectively analyze application principle and the clinical outcome of percutaneous MWA of the BBLs under ultrasound (US) guidance with a relatively large sample.

## MATERIALS AND METHODS

### Patient enrollment

This prospective study was approved by our institutional review board and medical records and imaging studies were reviewed. Written informed consent for the procedure was obtained from each enrolled patient. The study has been registered in Clinical-Trials.gov and the identifier number is NCT 02860104. From November, 2013 to April, 2016, a total of 122 patients diagnosed with BBLs by US-guided core needle biopsy in our hospital were recruited in this study and underwent US-guided percutaneous MWA at our department. Among them, 25 patients diagnosed by US or magnetic resonance imaging (MRI) as Breast Imaging Reporting and Data System (BI-RADS) category 4 and 97 patients as BI-RADS category 3. Information for each patient included demographics; longest diameters of BBLs; BBL numbers; BBL pathological type; location of the BBL according to whether adjacent to skin, pectoralis and areola. Ablation variables including session, puncture, time, and power; complications; volume reduction, palpability, and cosmetic satisfying outcomes were also measured and recorded.

### Pre-procedure evaluation

The eligibility criteria included the following: *(a)* the BBLs proved by using core-needle biopsy; *(b)* the BBL continually increasing during a half year follow-up; *(c)* the symptoms of local pain, discomfortable or compression considered probably relating to the mass of breast; *(d)* the patient with evidently psychological pressure due to the occurrence of the BBL despite clearly benignancy on imaging; *(e)* the patients unwilling or refusing to receive other treatment and presence of an appropriate route for percutaneous puncture under US guidance. The exclusion criteria included the following: *(a)* the patients who were pregnant or breast-feeding; *(b)* the patients with evidence of coagulopathy or acute or severe pulmonary insufficiency or heart dysfunction; *(c)* the patients during menstrual period; *(d)* the patients referring to other therapies including surgical excision and vacuum-assisted biopsy.

Prior to the procedure the number and location of masses were evaluated by conventional US and two kinds of contrast enhanced imagings (US and MRI). The maximum diameter of masses was measured at US, which was almost the same as assessed by using contrast enhanced US and MRI. The mass volume was obtained by multiplying the three diameters of the mass by 0.525 (ellipsoid volume). The data were extracted and analyzed by doctors J.Y. (10-year experience in interventional radiology), W.H. (3-year experience in radiology) and H.Y. (3-year experience in radiology). According to the relationship between the BBL and the adjacent tissue, BBL location was categorized as in the parenchyma and adjacent to the skin, the pectoralis or the areola (distance between BBL margin and these tissues less than 2 mm, which was measured on US images).

### US-guided MWA

US guidance was performed with a GE LOGIQ E9 scanner (R4; GE Medical Systems US& Primary Care Diagnostics, Wauwatosa, USA) with 9.0-5.0 MHz Convex array multi-frequency transducer. The microwave unit (KY-2000, Kangyou Medical, Nanjing, China) is capable of producing 100 Watts of power at 2450 MHz. The needle antenna has a diameter of 1.6 mm (16G) and a length of 10 cm. The active tip length is 3mm and 5mm. After local anesthesia with mixture of 2% lidocaine and 1% ropivacaine (1:1) subcutaneously and around the mass, the antenna was percutaneously inserted into the BBL and placed at the desired location under US guidance. For all the BBLs only one antenna was inserted along the long axis of BBL to perform ablation. When the BBL was less than 2.0 cm, a power output of 20 Watts and 3mm active-tip antenna was used. When the BBL measuring 2.0 cm or greater, a power output of 30 Watts and 5mm active-tip antenna was used. For the BBLs with the size less than 1.0cm, fixed applicator technique was used and for the > 1.0cm BBLs pull back technique was used as reported in thyroid ablation [14]. For the pull back technique, the applicator tip was initially positioned in the deepest portion of the BBL and ablation was then begun. The applicator tip moved slowly but continuously and when a heat-generated hyperechoic area was detected along the needle, the applicator tip was pulled back along the long axis of the needle until the tip arrived at the margin of mass. The applicator tip was then superficially re-positioned and then pulled along the long axis of the needle again until the hyperechoic area completely encompassed the entire BBL. At every applicator tip site, the microwave emitting duration was 10-30 seconds.

Hydro-dissection technique was used to auxiliarily ablate the BBLs adjacent to skin, pectoralis and areola. Before MWA, a PTC needle(HAKKO, Nagano, Japan) with a diameter of 0.7 mm (22G) and a length of 7 cm was inserted to the site between the margin of BBL and skin, pectoralis or areola under US guidance and 10-30ml saline were infused slowly for adjacent tissue protection during the whole ablation procedure.

### Follow up and imaging analysis

After MWA, conventional US and dynamic contrast enhanced US/MRI was performed to evaluate the treatment efficacy. US scan equipment was the same as ablation guidance equipment. US contrast agent was Sonovue (Bracco Company, Milan, Italy). Contrast enhanced MRI was performed using a 3.0-T unit (Signa Echo-Speed, GE Medical Systems, USA). MRI had the distinct advantage in evaluating the ablation zone [15], so all the patients performed MRI scan at 1-3 days after the ablation for therapeutic effect assessment.

If irregular peripheral nodular enhancement was noted, this was thought to indicate the presence of residual unablated BBL. Then further ablation was considered if the patient still met the criteria for MWA. In patients with complete necrosis, a well defined non-enhancing BBL on contrast enhanced MRI/US was noted, then routine US was repeated for its convenience and cheapness to monitor breast at 3, 6 months after MWA and then at 6-month intervals. If necessary, contrast enhanced MRI/US could be chosen to evaluate the new BBL and ablated BBL. The ablated BBL gradually decreased in zone over time.

Clinical symptoms and complication were also recorded during the follow-up. However, our central concern was the volume reduction ratio(VRR) which was calculated by the following equation: VRR (%) = [(initial volume - final volume) × 100]/initial volume. The ratio was assessed by US measurements pre-treatment and at the last follow-up visit.

### Statistical analysis

Continuous variables including BBL volume, ablation time, power and applicator insertion were compared between the subsets by Student’s *t*-test and Wilcoxon signed-rank test. One-way ANONA (analysis of variance) was used to examine the difference of the VRR between the ≤ 1.0cm, 1.1-2.0 cm, and > 2.0cm BBLs. Data were reported as mean ± S.D. or median. All statistical analyses were performed using the SPSS 16.0 for windows statistical package (SPSS, Chicago, IL) by doctors Y.J., W.H.. The difference with a *P* value of less than 0.05 was considered statistically significant.

## RESULTS

### Baseline characteristics

US-guided MWA was performed for 198 BBLs in 122 patients. Median follow-up was 14.0 (ranging 10.0-34) months. The period of observation for these patients was 6-12 months for 49 patients (40.2%%, 49/122), 12-24 months for 58 patients (47.5%, 58/122), more than 24 months for 15 patients (12.3%, 15/122). Indications for MWA of these 122 patients were the following: BBLs increasing obviously during past half one year in 25 patients, discomfortable symptoms probably relating to the BBLs in 9 patients, evidently psychological pressure due to the occurrence of the BBLs in 63 patients, the BBLs with BI-RADS category 4 diagnosed by US/MRI (but benign by pathology) in 25 patients. A total of 19 BBLs with the size less than 1.0cm were ablated for the reasons of 9 lesions with the imaging diagnosis of BI-RADS category 4 and 10 with evidently psychological pressure.

In the 122 patients, all the BBLs were successfully identified and ablated, and all the masses were ablated in one session even for the patients with bilateral lesions. The clinical data of these patients are shown in the Table 1. According to the growth location of BBLs, 27 were adjacent to skin, 22 adjacent to the pectoralis, 36 adjacent to the areola and 113 in the parenchyma. The BBLs could be palpable in 90.2 % (110/122) of the cases.

**Table 1:** The clinical features of patients and masses.

Variable	Datum
No. of patients	122
With 1 mass ablated^*^	71 (58.2%)
With 2 masses ablated^*^	35 (28.7%)
With 3 masses ablated^*^	11(9.0%)
With 4 masses ablated^*^	3(2.5%)
With 6 masses ablated^*^	2(1.6%)
Mean age(y)^†^	36.6±10.5 (17-74)
No. of masses	198
≤2 cm	156 (78.8%)
>2 cm	42 (21.2%)
Mean mass diameter(cm)^†^	1.6±0.7 (0.7-4.9)
Growth locations of masses	
Adjacent to skin^*^	27 (14.4%)
Adjacent to pectoralis^*^	22 (11.8%)
Adjacent to areola^*^ In the parenchyma^*^	36 (19.3%) 102(54.5%)
Median ablation time(min)^§^	3.2 (0.5-18.3)
Ablation power(W)^†^	28.3±6.2(20-50)
Mean ablation insertions^†^	1.2±0.4 (1-2)
Median follow-up(mo)^§^	6.0 (4-26)

Note.—* Data in parentheses are percentages.

^†^Data are means ± standard deviation; data in parentheses are ranges.

^§^Data in parentheses are ranges.

### Therapeutic response

**Figure 1:**
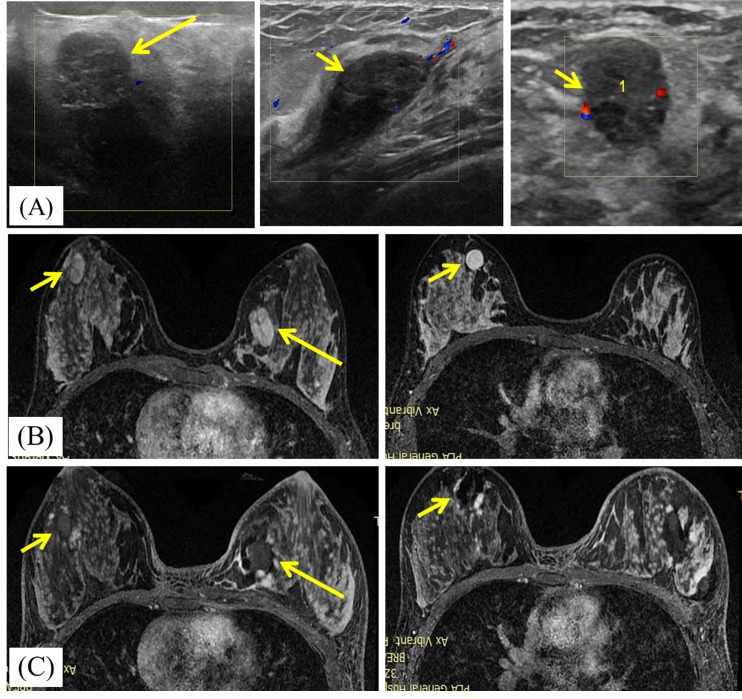
A 23-year-old woman with bilateral breast fibroadenomas **A.** Ultrasound scan before microwave ablation(MWA) shows the three hypoechoic BBLs with poor blood flow signal. The masses size are 2.8cmx1.3cmx1.5cm (in left breast, large arrows), 1.5cmx1.4cmx1.2cm (in right breast, small arrows) and 2.2cmx1.0cmx2.1cm (in right breast, small arrows), respectively. **B.** Transverse contrast-enhanced magnetic resonance imaging (MRI) shows hyperintensity masses in left breast (large arrows) and right breast (small arrows) before MWA in arterial phase. **C.** Contrast-enhanced MRI image shows hypointensity MWA treatment zone (arrows) for bilateral breast fibroadenomas in arterial phase.

All the patients received one session treatment with MWA for all the BBLs. The median duration to reach complete ablation at US was 3.2

minutes (ranging 0.5-18.3 minutes). The mean power of MWA was 28.3 ± 6.2W (ranging 20-30W). 85 patients received fluid infusion betweenthe BBLs and adjacent tissues. After MWA, the echogenicity of the treated BBL decreased gradually and became heterogeneous

hypoecho in about 5 minutes on US imaging. Non-enhancement was showed on contrast enhanced US and MRI after MWA (Figure 1)

### Technique success and volume reduction

**Figure 2:**
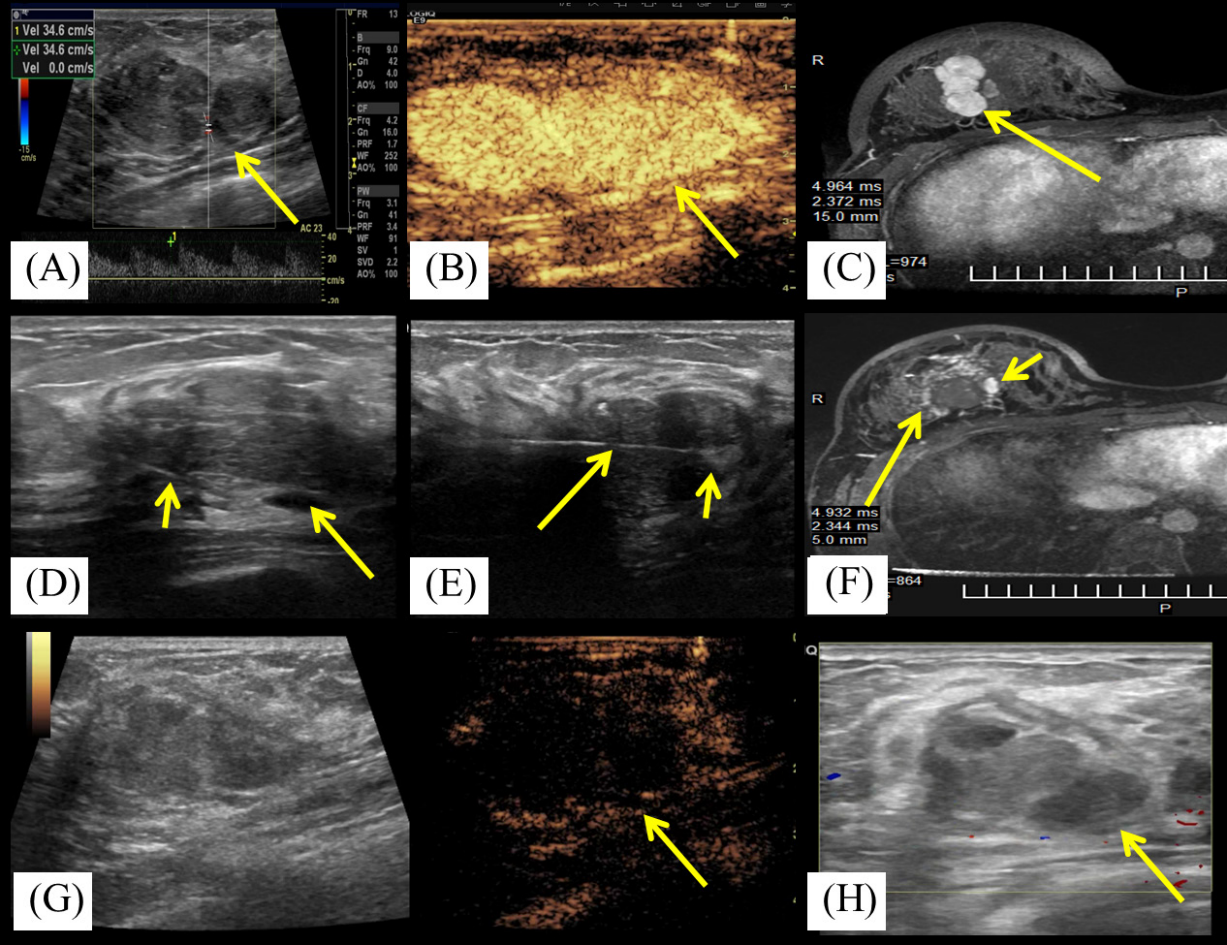
A 19-year-old woman with right breast fibroadenoma **A.** US scan before MWA shows the hypoechoic mass with arterial blood flow signal with the rate of 34.6cm/s. The mass is adjacent to the pectoralis and the size is 3.6cmx2.4cmx1.7cm. **B.** Contrast-enhanced US before MWA shows the mass is hyper-enhancement (arrow) in arterial phase. **C.** Transverse contrast-enhanced MRI shows hyperintensity mass in right breast (arrow) before MWA in arterial phase. **D.** US scan before MWA shows the fluid (arrow) infused by the fine needle (small arrow) between the mass and the pectoralis. **E.** US scan shows the centrally placed antenna (arrow) in the mass and increased echogenicity near the irradiating segment of the antenna (small arrow) at the beginning of MWA session. **F.** Contrast-enhanced MRI image shows hypointensity treatment zone (arrow) and the peripheral nodular enhancement (small arrow) in arterial phase at third day after MWA. **G.** Contrast enhanced US at second day after MWA shows the mass is non-enhancement (arrow) in arterial phase. **H.** US obtained 3 months after MWA shows the heterogeneously hypoechoic ablation zone without blood flow signal and with the size of 3.3cmx2.2cmx1.5cm.

Technique success referred to the BBL was treated according to protocol and was covered completely by the ablation zone. Based on US and contrast enhanced US imaging evaluation, technique success was achieved in 198 of 198 (100 %) BBLs. But MRI concluded technique success was achieved in 197 of 198 (99.5%) BBLs for one BBL with the size of 3.6 cm was shown incomplete ablation by MRI scan three days after MWA. The patient was performed another contrast enhanced US scan at 3 months after MWA and the ablated BBL showed non-enhancement with the largest diameter of 3.3cm (Figure 2).

**Figure 3:**
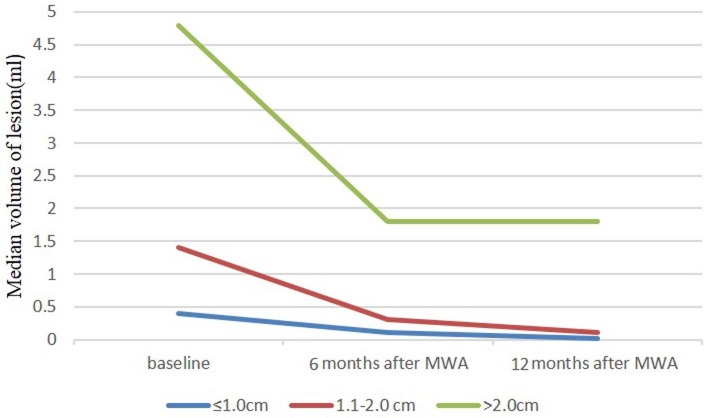
Mean volume of ≤ 1.0cm, 1.1-2.0cm and > 2.0cm nodules at baseline (time of MWA) and at follow-up after treatment

The changes in the volume of the masses before MWA and at each follow-up period are summarized in Table 2. Totally, the VRR were 64.3 ± 44.9%(ranging -229.7-99.8%) and 78.4 ± 33.5%(ranging -140.1-95.3%) at the 6-month and 12-month follow-up, respectively, which showed significant statistical difference (*P* < 0.001). There were 15 patients with 25 BBLs that achieved a 24-month follow-up. The baseline mean size of 25 BBLs was 1.8cm ± 0.8(ranging 0.7-4.4cm) and median volume was 2.6 ml (ranging 0.3-44.7 ml). The VRR were 50.8 ± 30.6% (ranging -138.5-100.7%), 63.4 ± 41.5% (ranging -140.8-106.7%) and 80.6 ± 31.7% (ranging -138.3-100.8%) at the 6-month, 12-month and 24-month follow-up, respectively, which showed significant statistical difference (*P* = 0.008). For masses with the size of ≤ 1.0cm, the VRR at the 6-month and 12-month follow-up were 71.4 ± 28.0% (ranging 32.7-100.7%) and 89.3 ± 20.8% (ranging -110.9-100.7%), respectively. For masses with the size of 1.1-2.0cm, the VRR at the 6-month and 12-month follow-up were 58.3 ± 42.9% (ranging -227.9-90.5%) and 84.7 ± 27.6% (ranging -30.9-100.1%), respectively. And for masses with the size of > 2.0cm, the VRR at the 6-month and 12-month follow-up were 45.3 ± 28.8% (ranging -20.5-90.7%) and 55.9 ± 32.9% (ranging -22.8-91.6%), respectively. The smaller masses showed a significantly better reduction than the larger masses (*P* < 0.001)(Figure 3).

**Table 2:** Mass characteristics and the effect on ablation procedure.

Variable	Baseline	Median time(min)	Power(W)	Insertion	6 months post ablation	12 months post ablation	*P* value
≤1 cm		3.0(0.5-8.0)	26.3±8.1	1.0±0.0			
No. of masses	33						
Median volume(ml)	0.4(0.2-0.5)				0.1(0.01-0.4)^*^	0.01(0.01-0.4)^*^	0.046
VRR(%)					71.4±28	89.3±20.8	
1.1-2.0cm		3.0(1.0-11.5)	28.5±4.7	1.1±0.3			
No. of masses Median volume(ml) VRR(%)	112 1.4(0.6-4.2)				0.3(0.1-2.6)^*^ 58.3±42.9	0.1(0.01-1.8)^*^ 84.7±27.6	0.001
>2.0cm		3.9(1.3-18.3)	30.5±8.2	1.5±0.5			
No. of masses Median volume(ml)	42 4.8(1.1-47.6)				1.8(0.5-31.1) ^*^	1.8(0.5-28.8) ^*^	0.13
VRR(%)					45.3±28.8	55.9±32.9	
*P* value							
Volume	<0.001	0.37	0.004	<0.001	<0.001	<0.001	
VRR^†^					0.006	<0.001	

Note.— Unless otherwise indicated, data are means ± standard deviation.

Data in parentheses are ranges.

*P<0.001when compared with baseline data.

^†^*P*<0.01 when compared between 1.1-2.0cm and >2.0cm masses and between ≤1 cm and >2.0cm masses, *P*>0.05 when compared between ≤1 cm and 1.1-2.0cm masses.

VRR; Volume reduction ratio.

### Cosmesis and satisfaction

Cosmetic outcomes with the skin texture, pigmentation and wound which were based on major or minor complications [16] were reported as excellent, good, acceptable, and poor. 110/122(90.2%) patients reported excellent cosmesis and 12/122(9.8%) patients reported good cosmesis. The major factor affecting the satisfaction results in this study was the presence of mass formation secondary to BBL coagulation necrosis at the MWA site. 118/122(96.7%) patients reported the ablated BBLs became softening gradually during the follow-up and 4/122(3.3%)patients reported the ablated BBLs were as the same hardened as those before ablation. 56/122(45.9%) patients reported the ablated BBLs were not palpable at all and 66/122(54.1%) patients reported volume reduction at the median follow-up of 14 months.

### Complications/safety

Overall, the safety profile of MWA therapy appears very good. Treatment was well tolerated. A mild sensation of heat and pain in the ablation site was experienced by most of the patients, whereas, no one claimed the procedure to stop. No tranquilize medicines were given before or after ablation. During and after the procedure, there was no major complication and other adverse effect occurred in all the 122 patients.

## DISCUSSION

Minimally invasion and cosmetic outcomes are the new treatment focus of surgery for breast. Desiring to avoid scars from the surgery, image-guided ablation as a minimally-invasive therapy becomes one of promising interventional method for BBLs [17]. However, most of researches on ablation of breast are focused on breast cancer therapy [18, 19]. To our knowledge, there is only very limited publications reporting the results of MWA for breast by Zhou *et al.* [11-13] and two results from microwave phased array thermotherapy for breast cancer with low complete necrosis [20, 21].

In many ways, the breast is the perfect organ for ablation for covered only by skin with no intervening structures, less vascular structure compared with liver and kidney, and imaged effectively with US. Breast is composed of complex anatomic structure of closely juxtaposed fat and glandular and fibrous connective tissues. Therefore, breast is more heterogeneous and breast masses are always surrounded by tissues with heterogeneous conductivity, which may produce interference on thermal ablation. However, Zhou *et al.* [22] have concluded that MWA achieved comparable extent of coagulation zone at the same time-power combination among muscle, liver and adipose tissue. The results indicated that MWA might be not influenced by the content of the tissue and was suitable for BBL ablation regardless of components of the breast.

Among several thermal ablation techniques, RFA plays a major role in breast mass therapy. However, tissue electrical and thermal conductivities are especially important during RFA. A previous study suggested active heating of MWA was less affected by background breast composition than conductive heating of RFA [10]. Results of MWA from Zhou *et al.* [11, 12] demonstrated MWA achieved complete mass necrosis rate of 95.0% for 1.0-3.0 cm (mean 2.0cm)size of cancer, while RFA was also mostly used to treat small breast cancers (mean size < 2.0cm) and could achieve complete ablation rate of 50-100% from the results of more than 30 researches [23]. Lower rates of complete ablation (46.2-73.0%) were reported in other ablation therapies [24-26], including cryotherapy, laser and HIFU treatment. For BBLs therapy, thermal ablation was mainly focused on fibroadenoma therapy (Table 3). Cryoablation shared the most reports but almost from10 years ago, which showed 73-99% of the BBLs presented volume reduction at 12-month follow-up [5, 27-35]. The numbers of publications on RFA, laser and HIFU for BBLs are relatively smaller and with limited efficacy results [36-40]. Ablation has many advantages compared with vacuum-assisted biopsy for BBLs, which is recommended to treat < 2.5cm BBLs and less than 3 lesions with the potential risk of bleeding, hematoma, and skin dimpling. Thermal energy of MWA can cover tumors conformally under precise imaging guidance, therefore it can avoid bleeding and normal tissue injury. Safe procedure provides the chance to treat more and larger tumors during one session. Our results with a large sample provided 99.5% of technique success by MRI evaluation. 80% of VRR was achieved for ≤ 2 cm BBLs and 50% of VRR was achieved for > 2 cm BBLs at 12-month follow-up. And MWA needed less ablation time compared with that of RFA, cryoablation and HIFU.

**Table 3:** Summary of five thermal ablation techniques for fibroadenoma.

Author	Year	Technique	No. of masses	Mean mass size(cm)	Ablation needle size(mm)	Ablation duration (mins)	Follow-up (Mons)	CA(%)	volume reduction (%)	Major complication(%)
Kaufman et al [27]	2002	Cryoablation	57	2.1	2.4	6-30	12	N/A	65	0
Kaufman et al[28]	2004	Cryoablation	66	2.0	2.4	N/A	12	N/A	87.3	0
Kaufman [29]	2004	Cryoablation	57	2.1	2.4-2.7	14.8±3.3	12	N/A	89	0
Edwards [30]	2004	Cryoablation	89	1.8	2.7	N/A	6	N/A	51	2(abscess)
Caleffi *et al*[31] ^*^	2004	Cryoablation	124	2.0	2.4-2.7	14.7-16.1	12	N/A	92	0
Kaufman *et al*[32]	2005	Cryoablation	37	2.1	2.4	14.3	30	N/A	99	0
Littrup *et al*[33]	2005	Cryoablation	42	4.2 cm^3^	2.4	<30	12	100	73	0
Nurko [34]	2005	Cryoablation	444	1.8	2.7	22	12	N/A	68-73	0
Hahn [5]	2013	Cryoablation	23	≤ 3	3.4	20	12	91.3	75-76	4.5(severe pain)
Golatta *et al*[35]^]^	2015	Cryoablation	60	1.2 cm^3^	3.5	44-74	12	N/A	93	0
Teh HS [6]	2010	RFA	2	2.3 and 3.0	N/A	10 and 14	6	100	N/A	0
Dowlatshahi K [6]	2010	Laser	2	1.9-2.6	2.1	15-25	96	N/A	40-50	0
Basu S[36]	1999	Laser	30	2.2 cm^3^	0.8	5	2	N/A	60-70	26.7(skin burn)
Yang BR[37]^*^	2015	Laser	19	0.78	0.8	1-2	32	N/A	73.7	10(skin burn)
Hynynen K[38]	2001	HIFU	11	1.9 cm^3^	N/A	45-120	6	73	31.6	0
Kovatcheva R[40]	2015	HIFU	51	3.89ml	N/A	118	12	84.3	72.5	0
Cavallo Marincola B[39]	2015	HIFU	12	2.65	N/A	57.2	3	N/A	50	0
Present study	2016	MWA	187	1.6	1.6	3.2	12	99.5	80	0

Note.— RFA, Radiofrequency ablation; HIFU, High-Intensity Focused Ultrasound; MWA, microwave ablation; CA, complete ablation; N/A, not available.

*study for benign breast lesions.

Our results suggest that as with other interstitial thermal ablation techniques, MWA can achieve excellent results in BBLs and is effect for the management of larger and “high risk” localized BBLs. Noticeably, in order to improve MWA efficacy and to minimize the complication, some important strategies needed to be noted: (1) As a common benign disease itself, strict ablation indications should be followed. And unnecessary or excessive treatment should be avoided. (2) Careful US scan and sometimes combination with MRI scan are necessary to identify the size, border and location of the BBL before ablation. (3) Breast tissue can be compressed asymmetrically and BBL is not easy to fix during percutaneous needle puncture. So it is difficult to place the ablation applicator at the center of the BBL as small liver cancer ablation. Moreover, lower microwave power of 20-30 W was used in breast to avoid thermal injury, so the ablation zone is limited at single emitting site to cover the whole BBL. Therefore, we suggest using pull back technique as used in thyroid [14] to ablate the BBLs. (4) If the BBL is close to skin, nipple, chest muscle, or breast implant, then hydro-dissection with saline or temperature monitoring should be planned. (5) Combined with multi-modality imaging including US, CEUS and MRI to accurate assessment of the ablation and the subsequent follow-up surveillance are necessary [41]. And breast MRI has shown greater sensitivity and better residual BBL detection, so for large, irregular and multiple masses, MRI scan is a good choice to evaluate the effect [15]. Our study has some limitations. Firstly, the number of patients with larger than 4 cm BBLs and with BBLs adjacent to important structures was relatively small. Further studies are needed to include larger BBLs and risk localized BBLs. Secondly, longer follow-up period is warranted to evaluate the long-term volume reduction efficacy for BBLs and the lactation influence on nulliparous patients. Lastly, the prospective multi-center data were expected to evaluate the clinical outcomes of the MWA for BBLs.

## CONCLUSIONS

In summary, our prospective results show US-guided percutaneous MWA yields effective mass kill in BBLs patients without causing complications. It is a safe and effective technique for the treatment of BBLs in selected patients. Large randomized controlled study is warranted to observe its treatment efficacy and compare the results with those of other ablation options.
